# Editorial: Development of Functional Foods From Marine Sources

**DOI:** 10.3389/fnut.2021.812497

**Published:** 2021-12-01

**Authors:** Jian Zhong, Enrique Barrajón-Catalán, Jose Manuel Lorenzo, Jun Lu, Brijesh K. Tiwari

**Affiliations:** ^1^National R&D Branch Center for Freshwater Aquatic Products Processing Technology (Shanghai), Integrated Scientific Research Base on Comprehensive Utilization Technology for By-Products of Aquatic Product Processing, Ministry of Agriculture and Rural Affairs of the People's Republic of China, Shanghai Engineering Research Center of Aquatic-Product Processing and Preservation, College of Food Science and Technology, Shanghai Ocean University, Shanghai, China; ^2^Instituto de Biología Molecular y Celular and Instituto de Investigación, Desarrollo e Innovación en Biotecnología Sanitaria de Elche, Universidad Miguel Hernández, Elche, Spain; ^3^Centro Tecnológico de la Carne de Galicia, Ourense, Spain; ^4^Área de Tecnología de los Alimentos, Facultad de Ciencias de Ourense, Universidad de Vigo, Ourense, Spain; ^5^School of Science, Faculty of Health and Environmental Sciences, Auckland University of Technology, Auckland, New Zealand; ^6^Department of Food Chemistry and Technology, Teagasc Food Research Centre, Ashtown, Ireland

**Keywords:** marine foods, functional active substances, functional foods, aquatic products, extraction techniques, emerging technologies

Ocean is a great treasure and can provide important food and nutrition resources for humans. With the increase in global population size, living standards and food demands of mankind, consumption, and demand of marine foods will continue to increase globally. Food production from the ocean is sustainable and has significantly lower environmental impact compared to terrestrial foods. Indeed, the ocean has potential to supply over 3 billion tons of marine food to feed 30 billion people. In addition, marine food raw materials can provide high quality sources of protein for human consumption. However, it is important to view the potential benefits of marine food consumption in the context of increasing concerns with ocean pollution (e.g., plastic debris, industrial waste) and its potential to damage marine life.

Research has shown that diets with increased consumption of marine foods are associated with reduced risk of diet-related chronic diseases. Marine foods are low in calories, and contain functional active substances such as bioactive peptides, terpenes, polyketides, alkaloids, unsaturated fatty acids, polysaccharides, vitamins and minerals, which have been shown to have positive health effects. As such, the extraction of functional active substances and the development of functional foods from marine resources is attracting increasing attention in the field of nutrition and health, and food science and technology.

To date, studies on marine foods have focused on three areas: (1) animal and plant-based sources of raw materials such as fish, shrimp, shellfish, and aquatic plants; (2) functional active ingredients such as bioactive peptides, unsaturated fatty acids, polysaccharides, vitamins and minerals which can be extracted from raw materials or their by-products; (3) preparation and development of functional ingredients such as fish oil emulsions, bioactive peptide powders, polysaccharide capsules, marine algae tablets, and shellfish-originated drinks. However, further studies related to sensory (e.g., taste) evaluation and molecular mechanisms and pathways of the different types of marine foods are important, to further advance discovery of new functional ingredients, product applications and nutritional and pharmaceutical therapeutics. In addition, with the increasing concern of ocean pollution and the risk it poses to marine sources, food safety aspects of marine foods (e.g., toxicity) also require further investigation.

With the development and application of novel and emerging processing techniques (e.g., microwave, radio frequency, 3D printing), extraction techniques (e.g., ultrasonic extraction, microwave-assisted extraction) and analytical technologies (e.g., omic techniques, fast detection techniques), there is the potential for significant advances in our understanding. It is envisaged that these advances will provide the knowledge required to promote and optimize the development of marine-based products for human consumption.

This special issue is a platform to discuss development of functional foods from marine sources. Yu et al. explored the effect of cold chain logistic interruptions on lipid oxidation and volatile organic compounds of salmon (salmo salar) and their correlations with water dynamics. Their work suggested that the temperature fluctuation during cold chain logistics should be avoided to maintain the nutrients and freshness of salmon. Ma et al. analyzed the chemical localization, structural change, and antioxidant property of the colloidal particles in tuna head soup. Their results provided basic information to understand the colloidal particle formation in food soup and suggested food soup might be a potential high-value-added way for aquatic by-product tuna head. Wu et al. characterized a food grade emulsion stabilized by the by-product proteins extracted from the head of giant freshwater prawn (*Macrobrachium rosenbergii*). Their work suggested a potential high-value-added way for prawn by-product. Li et al. explored the shelf-life extension of large yellow croaker (*Larimichthys crocea*) by active coatings containing lemon verbena (*Lippa citriodora* kunth.) essential oil. Their results found that the locust bean gum-sodium alginate active coatings incorporated with lemon verbena (*Lippa citriodora* Kunth.) essential oil emulsions maintained the quality and extended the shelf life of large yellow croaker during refrigerated storage. Jia et al. explored the effect of oral nutritional formula with three different proteins on type 2 diabetes mellitus *in vivo*. Their work provided a potential way to develop and evaluate marine protein-based nutritional products. Huang et al. reviewed Marine bioactive compounds as nutraceutical and functional food ingredients for potential oral health. Poulose et al. analyzed the anti-diabetic potential of a stigmasterol from the seaweed *Gelidium spinosum* and its application in the formulation of nanoemulsion conjugate for the development of functional biscuits. Their work demonstrated the stigmasterol could be used as a supplement in diets for diabetic patients. Aspevik et al. studied the nutritional and sensory properties of protein hydrolysates based on salmon (*Salmo salar*), mackerel (*Scomber scombrus*), and herring (*Clupea harengus*) heads and backbones. Their work showed only minor variations were found in the final protein hydrolysate products. Xu et al. reviewed the application of marine-derived collagen as biomaterials for human health. Their work pointed out that marine-derived collagen will attract more and more attention in the fields of clinical, medicine, food, etc. All these works proved ocean could provide excellent marine sources for the development of functional foods.

## Author Contributions

All authors listed have made a substantial, direct, and intellectual contribution to the work and approved it for publication.

## Funding

This research has been supported by research grants from the National Key R&D Program of China (No. 2019YFD0902003).

## Conflict of Interest

The authors declare that the research was conducted in the absence of any commercial or financial relationships that could be construed as a potential conflict of interest.

## Publisher's Note

All claims expressed in this article are solely those of the authors and do not necessarily represent those of their affiliated organizations, or those of the publisher, the editors and the reviewers. Any product that may be evaluated in this article, or claim that may be made by its manufacturer, is not guaranteed or endorsed by the publisher.

